# Multifunctional Milk-Derived Small Extracellular Vesicles and Their Biomedical Applications

**DOI:** 10.3390/pharmaceutics15051418

**Published:** 2023-05-06

**Authors:** Youxiu Zhong, Xudong Wang, Xian Zhao, Jiuheng Shen, Xue Wu, Peifen Gao, Peng Yang, Junge Chen, Wenlin An

**Affiliations:** 1 Wenlin An’s Laboratory, National Vaccine & Serum Institute (NVSI), China National Biotech Group (CNBG), Sinopharm Group, No. 38 Jing Hai Second Road, Beijing 101111, China; 2 Beijing Advanced Innovation Center for Biomedical Engineering, School of Engineering Medicine & Shenzhen Institute of Beihang University, Beihang University, Beijing 100083, China

**Keywords:** milk-derived small extracellular vesicles (msEVs), small extracellular vesicles (sEVs) engineering, isolation and purification, drug delivery, biopharmaceutical applications

## Abstract

In recent years, small extracellular vesicles (sEVs) have been regarded as the next generation of novel delivery systems after lipid nanoparticles because of their advantages and huge prospects in drug delivery. Studies have shown that sEVs are abundant in milk and therefore can be a large and economical source of sEVs. Natural milk-derived small extracellular vesicles (msEVs) have important functions such as immune regulation, anti-bacterial infection, anti-oxidative, etc., and play a beneficial role in human health at multiple levels, including intestinal health, bone/muscle metabolism, and microbiota regulation. In addition, because they can pass the gastrointestinal barrier and have low immunogenicity, good biocompatibility, and stability, msEVs are considered a crucial oral drug delivery vehicle. Moreover, msEVs can be further engineered for targeted delivery to prolong the circulation time or enhance local drug concentrations. However, msEVs separation and purification, complex contents, and quality control hinder their application in drug delivery. This paper provides a comprehensive review of the biogenesis and characteristics, isolation and purification, composition, loading methods, and function of msEVs, based on which their applications in biomedical fields are further explored.

## 1. Introduction

sEVs are biological nanovesicles that range in size from 30 to 150 nm, and cells continuously secrete or accept sEV from other cell sources [1]. Almost all cells are capable of secreting sEVs in normal or abnormal physiological states [2], which are present in the fluids of the eukaryotic body (e.g., saliva, blood, urine, amniotic fluid, etc.), plant juices, and supernatants of cultured cells, and different environmental factors and health conditions can affect the composition of sEVs [3,4,5]. Among these sources, milk is utilized with great interest due to its large yield, easy availability, and high sEVs content, which endow it with great potential in clinical applications. In milk, sEVs play significant roles in immune defense, growth, and physiological regulation via nucleic acids, proteins, sugars, etc. [6,7,8,9]. Being different from the other sEVs from body fluids, milk sEVs stay the same upon subjecting them to highly acidic conditions as well as digestive enzymes in gastroenteric environments. 

Nowadays, sEV biomarkers include CD9, CD63, CD81, TSG101, HSP70, etc. [10,11,12,13]. sEVs are regarded as a crucial communication intermediary for cells [14]. Donor cells are capable of transferring important information or functional substances (e.g., proteins, nucleic acids, and lipids) to responsive cells through sEVs. Information and substance delivery by sEVs play a crucial role in several biological activities [15,16]. Thus, sEVs can be utilized to assess normal physiological changes or mechanisms of disease onset and progression. In addition, sEVs can be used as biomarkers of a variety of diseases for disease detection.

Over the past decades, nano-drug delivery systems have made significant progress, including various formulations for biomolecular and chemical drugs [17,18,19]. Nevertheless, two challenges, such as poor biocompatibility and short blood circulation, need to be overcome for these delivery systems before practical applications [20]. Due to their unique natural properties, sEVs are considered the next generation of highly promising delivery vehicles after lipid nanoparticles [21]. CD47, which is present on the surface of sEVs, prevents recognition by macrophages, therefore facilitating the prolongation of sEV circulation in the blood. It is demonstrated that the integrity of msEVs bilayers can be sustained in vitro for a long time, even under strong acid in the stomach and degradation in the intestine. Furthermore, they can reach the target tissue through the biological barrier. As a member of the sEVs family, msEVs are considered potentially excellent drug vehicles, especially the oral route, in terms of stability, toxicity, and cost [22].

**Figure 1:**
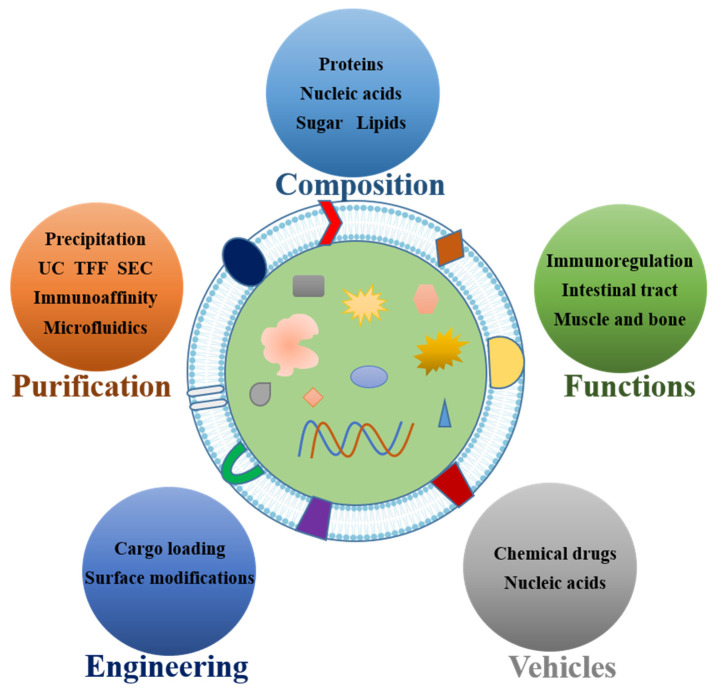
Overall framework of the full text.

This review summarizes the important and advantageous implications of msEVs on human health from several perspectives. A comprehensive outlook on the future functional and application development of msEVs is provided by summarizing the strategies related to the purification and drug loading of msEVs. Moreover, new forms of sEV applications, such as engineering modifications, are also reviewed. On this basis, we analyze the challenges faced in the application development of msEVs. The outline of the review is shown in Figure 1.

## 2. Biogenesis and Characteristics of sEVs 

Extracellular vesicles are categorized according to the biosynthesis or release pathway: sEVs have a diameter of 30–150 nm and are generated from the endocytic pathway; from the plasma membrane, particulate/micro-vesicles are released immediately and are about 100–1000 nm in diameter; apoptotic vesicles are about 50 nm–2 μm in diameter and are generated by apoptosis. The current most hotly studied subgroup is the sEVs.

**Figure 2:**
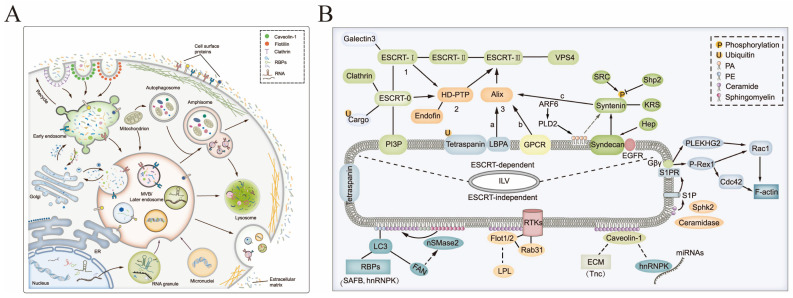
Overview of the process for sEV biogenesis (**A**) and multiple mechanisms (**B**); Copyright 2022, Springer Nature [32].

sEV biogenesis is a strictly controlled procedure consisting of three distinct stages: endocytic vesicle formation through plasma membrane invagination, multivesicular body formation through endosomal growth, and plasma membrane fusion, which results in the release of vesicles from multivesicular bodies, as shown in Figure 2. sEVs formation relies upon the endosomal sorting complex required for transport (ESCRT) and some GTP-binding protein-directed mechanisms [23,24,25]. In the early stage, the plasma membrane develops inward to give rise to the early endosomes, which change into mature late endosomes by degrees. In this process, material from the cell is selectively absorbed into luminal vesicles in the endosome, controlled by the ESCRT mechanism [26]. However, it has been demonstrated that sEVs can form in the absence of important components of four ESCRT complexes, suggesting that there are other modes of sEV formation [27]. In addition to the ESCRT protein family, lipids and several other proteins, such as lactadherins, platelet-derived growth factor receptors, membrane associates, microsomal proteins, GTPases, HSP, and quadruple transmembrane proteins, also participate in the biological process that produces sEVs [28,29,30]. It has been proven that lipids play a crucial role in vesicle biogenesis and transport, such as membrane deformation, fission, and fusion [31].

SEVs protein composition is inextricably linked to the type of cell or tissue from which they come. sEVs typically contain fusion proteins and membrane transport, and most sEVs have integrins, HSP, ALIX, members of the transmembrane 4 superfamily, and the Ras-associated protein GTPases Rab. The surfaces of both colostrum and msEVs contain surface markers: four transmembrane proteins (including CD63, CD9, CD81, and TM4SF), milk fat globule epidermal growth factor 8, raft-associated proteins, and internal signature proteins such as TSG101, ALIX, and HSP70 [33,34]. The endosomal sorting complexes or additional related factors such as TSG101 and ALIX participate in sEV genesis and can therefore be detected. All of these can be utilized as trustworthy sEVs identification markers, as shown in Table 1. sEVs do not contain p-selectin, integrin-β1, CD40, or calnexin, which are thought to be markers of vesicles of other origin. Differences in sEV compositions, especially in surface proteins, have a great influence on the recipient cells. sEVs surface expression of specific markers makes it possible to purify and identify specific groups of sEVs.

sEVs often require uptake by receptor cells to exert their function. MsEV trafficking in Caco-2 cells, human vascular endothelial cells, and IEC-6 cells is mediated by endocytosis [35,36]. This endocytosis-mediated msEVs uptake is dependent on cellular and msEVs surface glycoproteins in the rat and human intestines [35]. Due to the metabolic function of the liver, sEVs or msEVs accumulate mainly in the liver.

## 3. Isolation and Purification of msEVs 

How to obtain large amounts of high-purity sEVs is the primary problem to be solved to expand their application. Although many easy-to-use commercial kits have been developed, the generation of sEVs produced from milk on a huge scale for clinical use cannot be fundamentally solved by kits. The common purification methods for msEVs are comparable to traditional sEV purification techniques, including polyethylene glycol (PEG) precipitation, ultracentrifugation, density gradient centrifugation, ultrafiltration centrifugation, immunoaffinity capture, size-exclusion chromatography (SEC), and microfluidic techniques [54,55,56], as shown in Table 2.

### 3.1. Common Methods for sEV Purification

PEG precipitation purification is a method of coagulation and precipitation of large molecules of solutes caused by the addition of very hydrophilic PEG at specific salt concentration conditions and is commonly used for the isolation of proteins, nucleic acids, viruses, etc. sEVs have similar size and physicochemical properties to viruses. Therefore, PEG co-precipitation can be used to isolate sEVs, and PEG precipitation is also a commonly used method for commercial kits [57]. By employing this strategy, sEVs could be obtained with filtration or low-speed centrifugation without the need for special equipment. However, the purity of sEVs is low on account of coprecipitation proteins and polymers.

Currently, the most commonly used approach for purifying sEVs is ultracentrifugation [53]. The steps of low-speed centrifugation and high-speed centrifugation are mainly used in sequence: low-speed centrifugation can be used to separate milk’s floating fat and some free proteins, while ultracentrifugation is used to separate the majority of the cell waste and free proteins. Finally, clear jelly-like sEV aggregates can be observed in the centrifuge tube’s bottom, and sEVs of uniform size are gradually separated [58,59]. The current gold standard for sEV purification is ultracentrifugation. However, the purity of sEVs obtained by ultracentrifugation is still not guaranteed, and the sEV structure may be disrupted by repeated centrifugation. In order to improve their purity, density gradient centrifugation is carried out by layering a decreasing concentration of sucrose solution or iodixanol solution. sEVs are enriched mostly in the density range of 1.13–1.21 g/mL when subjected to a particular centrifugal force. Using this method, sEVs can be separated with high purity. Izumi et al. separated bovine msEVs by ultracentrifugation [58]. In order to improve their purity, the msEVs were washed twice to remove impurities such as proteins and lipids. By ultracentrifugation, proteins and lipoproteins in msEVs can be removed. In addition, Linares et al. pointed out that high-speed centrifugation leads to sEV aggregation, which can affect subsequent data processing [50]. Furthermore, the collection time is relatively high and the process is tedious, which limits the practical application of isolating sEVs on a large scale [44].

With a specific molecular weight cutoff, sEVs are separated by ultrafiltration centrifugation through an ultrafiltration membrane. sEVs relative molecular weight differs significantly from other impurities, and the use of membranes with appropriate molecular mass cutoffs allows the separation of sEVs from other biomolecules. Lobb and his colleagues have compared ultracentrifugation and ultrafiltration for sEV isolation, demonstrating that the isolation time of ultrafiltration (20 min) was much less than that of ultracentrifugation (180 min) [46]. Additionally, the above strategy has no influence on the biological activity of sEVs [61]. Nevertheless, clogging is observed on the membrane surface during the ultrafiltration separation process, leading to a reduction in the lifetime of membranes and low isolation efficiency. The clogging of the membrane surface is solved by tangential flow filtration (TFF). The flowing liquid is oriented perpendicular to the filtered liquid in a manner that is less likely to cause impurities or target product buildup on the membrane surface. Commonly used in the form of hollow fiber tubes and membrane packages, it is capable of separating and purifying biomolecules quickly and efficiently and can be used in many biological applications. Tangential flow devices have a number of drawbacks, such as high cost, significant shear stress, and difficulties working with small quantities [62].

The sEVs obtained by the above methods still have some room for improvement [61]. Kun Huang et al. found that increasing size exclusion chromatography (SEC) after superionization can further improve the purity of sEVs [67]. SEC isolates solutes due to the correlation between the sample’s particle radius and the pore size in the particles of the purification column. When impurities with smaller particle sizes enter the purification column, they will be temporarily intercepted, and the overall moving distance is longer. The sEVs do not enter the pores and are able to travel faster through the purification column; thus, they can be isolated and purified. The sEVs enriched by SEC technology are structurally intact, highly pure, retain strong functional activity, are simple to operate, and have good reproducibility [63,64,65].

Affinity purification is a purification method that uses specific binding interactions between molecules, such as specific binding between antigen and antibody. sEVs have several specific marker proteins on their surface, and magnetic beads with specific antibodies can be used for their capture. Surface markers vary between different sEV types and allow the isolation of different sEV isoforms. Immunoaffinity capture ensures the sEVs morphological integrity. It is highly specific, simple to perform, and does not require expensive instrumentation. However, this method is largely marker dependent and requires specific immunomagnetic beads, resulting in high cost and low benefit [66].

### 3.2. Large-Scale Purification of High-Purity msEVs

While the above methods for purification of sEVs are largely adequate for research applications of sEVs, higher purity is often required for commercialization and clinical use of sEVs. It is more difficult to scale up sEV extraction from milk than from cell supernatants.

Various types of extracellular vesicles and proteins are present in milk, making it a complex fluid. There is a high concentration of proteins in milk (about 3.5%), and most of them exist in aggregated form [68]. sEVs separation can be interfered with by some proteins mixing with them. sEVs separation may be hampered by the mixing of some proteins with sEVs. Casein forms micelles that are comparable in size to sEVs and can be co-separated with sEVs. Moreover, protein phosphorylation is one of the most important interference factors. The majority of the casein in milk has undergone phosphorylation and has a surface charge state resembling the phospholipid bilayer structure of sEVs [69]. Consequently, initial pre-treatment of milk is critical for sEV extraction and affects the purity and quantity of the separated sEVs.

Therefore, individual purification methods often fail to completely isolate high purity sEVs. The use of tandem methods for advantageous complementary purification of sEVs in milk becomes the preferred option. The general purification process is divided into three steps: pre-treatment of precipitated milk using acids, salts, and PEG, combined with techniques such as centrifugation, ultrafiltration, and tangential flow to remove a large number of heteroproteins such as casein, and further using size exclusion, well-established commercial Core700, qEV, and other methods to obtain high-purity msEVs [70,71,72,73,74,75], as shown in Figure 2. The combination of the above methods and the introduction of other new technologies make it possible to produce high-purity msEVs on a large scale and to achieve good batch stability and reproducibility. 

## 4. Composition and Functions of msEVs

**Figure 3:**
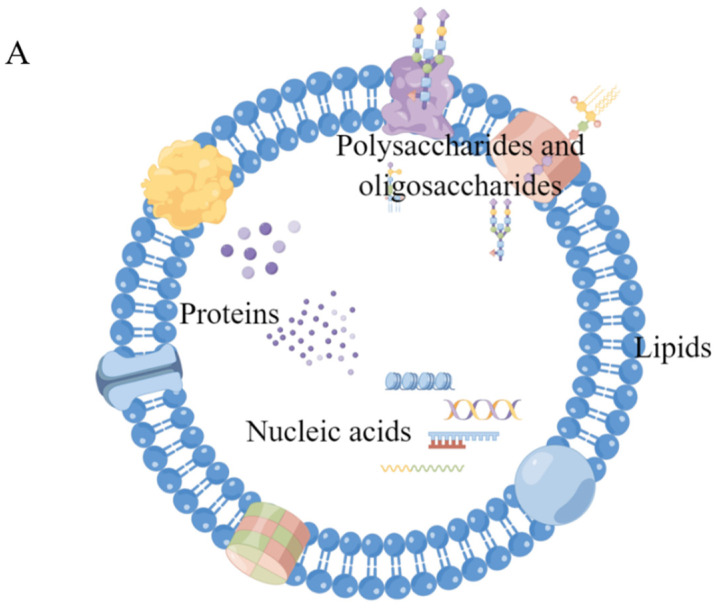

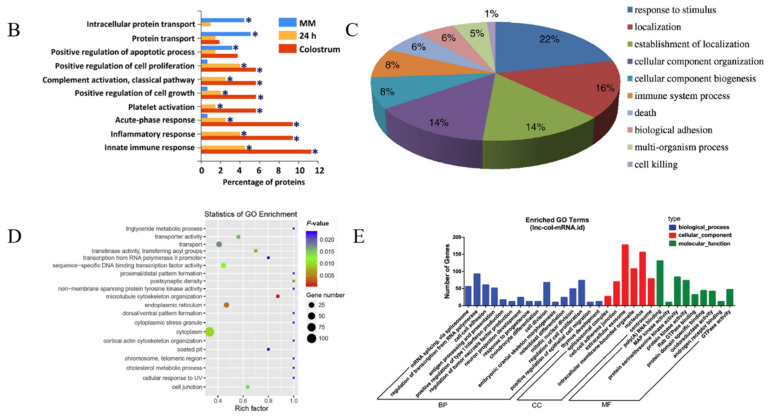
Composition and functions of msEVs. (**A**) Schematic of the composition of msEVs (by Figdraw, www.figdraw.com (accessed on 10 April 2023)) (**B**) Functional enrichment analysis using FunRich revealed msEVs from colostrum are enriched with proteins implicated in immune response. * *p* < 0.05; Copyright 2017, Springer Nature [48]. (**C**) GO annotation for msEV proteins in biological processes; Copyright 2017, Elsevier [77]. (**D**) The 20 most enriched GO terms for the circular RNA host gene; Copyright 2019, Elsevier [78]. (**E**) Gene ontology (GO) annotation analysis of bovine msEV long noncoding RNA (lncRNA); Copyright 2019, Elsevier [79].

Although the substances contained in sEVs from different sources have some commonalities, they are more often highly individual. Similar to sEVs isolated from other sources, sEVs from milk are rich in proteins, nucleic acids, sugars, lipids, etc., and many components are often unique to them [59,76], as shown in Figure 3.

### 4.1. Composition of msEVs

#### 4.1.1. Proteins

Proteins are important substances for the physiological functions of living organisms, and msEV proteins are essential for physiology and pathology. To date, the composition of bovine milk proteins has been reported to have changed in more than 300 articles depending on season, lactation period, breed, and health status, and it is inferred that the composition of msEVs varies accordingly.

The physiology and immunology of the host are revealed by sEVs proteins in milk, and between uninfected and infected cattle, there are 118 proteins that are expressed differentially and are engaged in many biological processes, including cellular processes, metabolic processes, and catalytic activities [80]. Using quantitative proteomic analysis, it has been demonstrated that the sEV load and that sEVs made from colostrum contain more proteins related to immune response, antimicrobial peptides, inflammatory response factors, cell growth, and complement activators than mature msEVs, which are rich in transport and apoptosis-related proteins [48].

The main milk lipoglobulin membrane proteins (Xanthine oxidase, Butyrophilin, Lactadherin, and Adipophilin) are the most prevalent proteins present in msEVs [81]. msEVs proteins are involved in multiple KEGG pathways, including leukocyte transendothelial migration, glycolysis/gluconeogenesis, amino acid-tRNA biosynthesis, galactose metabolism, the pentose phosphate pathway, and fatty acid biosynthesis [77]. Four abundant milk proteins, lactose anhydride, butyryl protein, periplasmin-2, and xanthine dehydrogenase/oxygenase, occur in the top 20 sEVs generated from cow’s and human’s milk [82]. The proteomes of BMEC-sEVs demonstrate their participation in the production of milk, suggesting that they may be one of the sources of msEVs [83]. The literature-based manual management online open database BoMiProt (http://bomiprot.org (accessed on 10 April 2023)) of the bovine milk proteome covers more than 3100 proteins from fat globule membranes, whey, and sEVs [84].

MsEV protein profiles provide important information in the area of newborn nutrition and health, providing novel insights into the composition of human and cow’s milk. The above information provides us with knowledge of bovine and human msEVs and may point to potential directions for the development of neonatal formulas, functional foods, and biomarkers.

#### 4.1.2. Nucleic Acids

Nucleic acids are among the most basic substances that constitute life and perform genetic information delivery and other physiological functions, containing miRNA, mRNA, and lncRNA that play crucial roles in the development of both physiological and pathological processes. Despite the high RNase activity in milk, there are roughly 1000 ng and 1700 ng of total RNA in the microvesicles that were extracted from 6 mL of mature and colostrum milk, respectively, and the majority of this RNA is miRNA [85]. The results showed that msEVs had higher levels of RNA under 200 nt than separated sEVs’ supernatant [85]. Numerous immune-related miRNAs can be found in msEVs. Colostrum had higher levels of immune-related miRNA expression compared to mature milk [86,87]. One study discovered 245 miRNAs in raw milk, and each miRNA can be dramatically changed at various lactational stages [88]. It is interesting that the expression of seven miRNAs remained relatively stable over the course of lactation [88]. 

In sEVs generated from milk, a substantial number of mRNAs were discovered. Most of the whey’s mRNAs were discovered in sEVs, according to a microarray study, while miRNAs in whey were found in supernatant and sEVs [58,85]. In the sEVs of milk and colostrum, mRNAs coding for CD36, FAS, Ea1, MHC-II, and MFG-E8 were proven to be present by RT-PCR [33]. 

A large number of lncRNAs are also present in bovine msEVs. lncRNAs regulate immune function, cell-cell communication, neurodevelopment, cell proliferation, reproduction, and osteoblastogenesis. lncRNAs are differentially expressed during different stages of lactation [79]. Digestion experiments have shown that bovine msEV lncRNAs are resistant to decomposition with several digestive fluids, such as pancreatic juice, gastric juice, bile, and saliva, in vitro [79]. Since they are packaged in membranes and the msEVs are extremely stable, the RNAs in them can be absorbed by the receptors to continue their functional effects [58].

The processing of milk also affects the content of nucleic acids in sEVs. The presence of ncRNA (non-coding RNA) in sEVs from seven commercial dairy products obtained at various processing phases was examined in one study, which demonstrated that processing had a substantial impact on ncRNA expression values [89]. Ultrasound treatment of milk also reduced the content of sEVs and miRNAs in milk [90].

#### 4.1.3. Polysaccharides and Oligosaccharides 

Milk oligosaccharides are thought to provide a natural way to alter key aspects of innate immunity in newborns. Studying oligosaccharides in msEVs provides greater insight into the interactions between breastfeeding and mucosal immune system development in infants. msEVs transport is a very important way for oligosaccharides from milk to reach the recipient cells [91]. Yet, little has been known about the oligosaccharides in msEVs, or “free sugars”.

The sEVs protein glycosylation study is a comprehensive tool for understanding the targeted delivery mechanism. One study analyzed the O-glycans, N-glycans, and free oligosaccharides of msEVs and whey by multiple derivatization strategies and identified 114 glycoproteins, most of which were located in sEVs [92]. These studies have contributed to our understanding of the biological and functional roles played by glycans in sEVs.

#### 4.1.4. Lipids

Although many dairy products are skimmed, the lipids in milk are still considered to be of great nutritional value. For example, milk phospholipids have a significant impact on infant intestinal and brain development [93]. MsEVs, like other sEV sources, are vesicles enveloped by double-layer lipids that secrete into milk through a different secretion mechanism from fat globules. Lipids are mainly composed of a unique mixture of polar lipids. Although there are different mechanisms involved in the formation and release of EV, they all need to be driven by the cooperation between lipids and proteins [94].

sEVs are rich in sphingolipids, cholesterol, and phosphatidylserine, and their content is two to three times that of donor cells [95]. Research has shown that the cholesterol content in msEVs accounts for 43.6% of the total lipid extract, which can increase membrane rigidity. There is an interaction between cholesterol and phospholipids in the sEV membrane, which causes the bilayer lipid membrane to be in a liquid-phase ordered state. The membrane fluidity of msEVs is slightly higher than that of liposomes composed of similar components [96]. Lipidomic analysis of msEVs would be more useful to understand the reasons for the superior stability of msEVs compared to other sources of sEVs.

### 4.2. Function of msEVs

**Figure 4:**
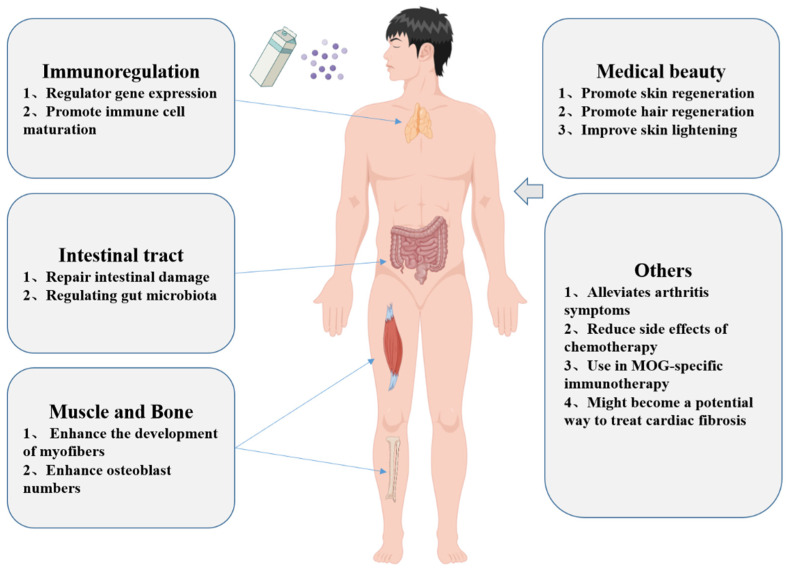
Functions and applications of msEVs (by Figdraw, www.figdraw.com).

After oral administration, the msEVs pass through the gastrointestinal barrier and reach the whole body through the circulation of blood and other bodily fluids. Macrophages, intestinal cells, and vascular endothelial cells all take up msEVs, which are then transferred to surrounding organs [36]. After the sEVs reach the receptor cells, some bind to the cell surface through protein-protein or receptor-ligand interactions to start the signal cascade and activate the endocytosis pathway. Some cells ingest sEVs through phagocytosis, phagocytosis, and fusion [97,98]. In vitro cell experiments or in vivo animal experiments have exhibited that msEVs can enter the cytoplasm through endocytosis and release their contents, such as miRNAs [76,99]. These studies indicate that sEVs in milk can be absorbed in vitro and in vivo, thus performing physiological functions. The physiological function of the sEVs in milk is multifaceted, as shown in Figure 4 and Table 3.

#### 4.2.1. Immunoregulation

MsEVs have significant impacts on metabolism and immune processes. Milk is rich in a variety of immune-related factors, and sEVs affect intercellular communication by binding to target cell receptors through surface antigens or by delivering sEVs RNA and proteins to target cells [100].

An enzyme called DNA methyltransferase 1 (DNMT1) is important in controlling epigenetic processes. MiRNA-148a, found in msEVs and milk fat globules, attenuates DNMT1 expression [106]. Kosaka and coworkers have reported that miRNA delivered by sEVs in breast milk could facilitate the immune system’s development in infants [141]. The number of Foxp3+ CD4+ CD25+ regulatory T cells in infants was increased in the presence of both miRNA-181a and miRNA-17, which are transported by sEVs. Inducing regulatory T cells is beneficial not only for healthy individuals but also for infants. Furthermore, T-cell-regulating miRNAs such as miR-181 and miR-155 were also rich in msEVs, which were beneficial for the differentiation of B-cells. As genetic material, miRNA could be transferred from mother to infant, supporting the development of an infant’s immune system and preventing a lot of infections. Similarly, Pieters et al. have demonstrated that sEVs in commercial milk have immunoregulatory cargo, including miRNAs and TGF-β, which could induce the differentiation of Th17 cells [104]. The results confirmed that the milk recipient’s immune system could be regulated by msEVs. 

#### 4.2.2. Regulation of Intestinal Tract Function

MsEVs have been validated to exert vital roles in the digestive tract [107,108]. Bovine msEVs possess protection against H_2_O_2_-induced oxidative stress. Evidence revealed that IEC-6 pretreatment with msEVs could enhance viability, increase GPX and SOD activity, reduce H_2_O_2_-induced LDH, ROS, and MDA accumulation, increase the amount of intracellular miR-155 and miR-146a, and inhibit H_2_O_2_-induced elevated Nrf2 and Ho1 gene expression [109]. In IEC-6 cells, msEVs can attenuate H_2_O_2_-induced alterations in purine metabolism and energy state and reduce purine nucleotide catabolism so as to exert their protective effects against oxidative stress [110].

The intestinal epithelial cell is an essential intestinal barrier, and its function will be impaired by hypoxia induction. Damage to the intestinal barrier can be prevented by msEV miRNAs in lactation. Among those miRNAs, bta-miR-34a is a potent regulator of mitigating hypoxic damage in IEC-6 [111]. sEVs made from yak milk have been shown to promote the growth of intestinal epithelial cells under hypoxia and activate protective signaling pathways, thus strengthening IEC-6 cell survival, and the protective effect is better than that of bovine msEVs [86]. As observed, IEC-6 cells ingested more msEVs generated from yak milk, which may be due to higher membrane protein expression [86].

**Figure 5:**
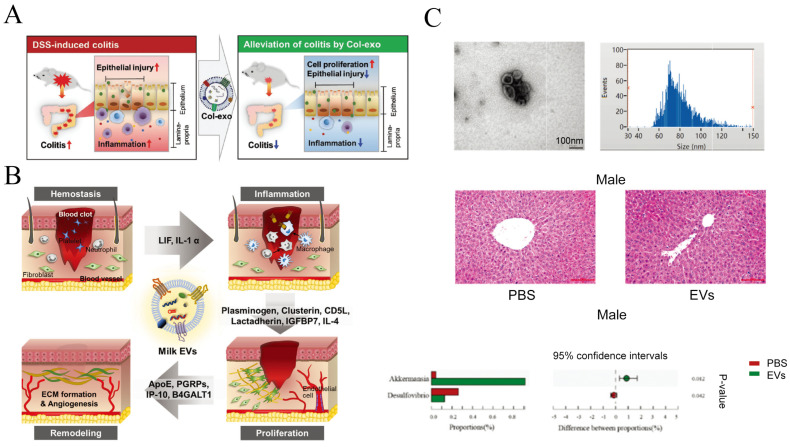
MsEVs have protective functions in the intestinal tract and promote skin regeneration. (**A**) Overall schematic diagram and characterization of the colostrum-derived sEVs; Copyright 2022, Royal Society of Chemistry [116]. (**B**) Schematic diagram of milk EVs-mediated cutaneous wound healing; Copyright 2021, John Wiley and Sons [128]. (**C**) Oral msEVs have positive effects on the intestinal microbiome and serum metabolism in mice; Copyright 2021, Royal Society of Chemistry [142].

The function of intestinal epithelial cells can also be impaired by other damaging factors, such as lipopolysaccharides. TLR4, which contributes to the occurrence of intestinal inflammation and necrotizing enterocolitis (NEC), can be inhibited by msEVs [112]. In a mouse genetically engineered to have ulcerative colitis, msEVs could prevent severe ulcerative phenotypes and intestinal inflammation [113]. Oral administration of msEVs can prevent NEC as approved by reducing morphological damage and injury to the ileum and reducing the number of MUC2+ and GRP94+ cells [114]. MsEVs enhanced the activity of gastric goblet cells and prevented experimental necrotizing small bowel colitis [115]. MsEVs can prevent dextran sulfate sodium-induced enterocolitis by inhibiting inflammation and oxidative stress, especially in colostrum-derived msEVs [116], as shown in Figure 5A. MsEVs altered the characteristic cuboidal form of normal colonic epithelial cells into a mesenchymal-like morphology and induced proliferation, which had not been seen in tumor cells [117]. The mechanism was attributed to the fact that msEVs up-regulated type I collagen expression and down-regulated Twist1 gene expression and PTEN proteins. sEVs derived from yak milk attenuate LPS-induced intestinal inflammation by suppressing activation of the PI3K/AKT/C3 signal pathway [118]. Severe acute malnutrition (SAM) is usually caused by epithelial atrophy and intestinal barrier disruption, and sEVs isolated from milk ameliorate malnutrition-induced intestinal barrier dysfunction [119]. 

Furthermore, msEV proteins and miRNAs were capable of preventing colitis through attenuating inflammation [123]. Upon subjecting cells to msEVs, TLR4-NF-κB as well as the NLRP3 signaling pathway in cells were inhibited, resulting in a reduction of the inflammatory response. In the inflamed colon, the disorder of cytokine production was corrected, and the balance between Th17 and Treg cells was also balanced. Additionally, msEVs could restore gut microbiota in ulcerative colitis, thereby modulating intestinal immunity. Therefore, the supplementation of milk sEVs in the daily diet was essential to maintaining the normal physiological function of the gut. Studies have demonstrated that dietary sEV deficiency leads to changes in the bacterial community of young rats and damages their intestinal health. sEVs generated from milk administered orally boosted the expression of GATA4, Muc2, MyD88, and RegII-g, all of which are associated with intestinal immunity [120]. Changes in the cecal bacterial community in C57BL/6 mice induced by consumption of msEVs [121]. Oral msEVs have positive effects on the intestinal microbiome and serum metabolism in mice [142], as shown in Figure 5C. The intestine is closely related to many inflammatory diseases. Systemic inflammatory illnesses like lupus erythematosus, multiple sclerosis, and rheumatoid arthritis can cause increased intestinal permeability, an unbalanced microbiota in the gut, and intestinal inflammation. Intervention of the intestinal tract, particularly the microbiome, by msEVs is expected to be a therapeutic measure for these diseases [122].

#### 4.2.3. Regulation of Muscle and Bone Development

MiRNAs from msEVs can alter the growth and development of muscle and bone, such as miR-21 and miR-29a, which can amplify mTOR signaling [124]. In mice, it has been noted that msEVs enhance osteoblast numbers and promote bone formation and osteoblast differentiation [125]. MsEVs enhance the development of myofibers in mouse C2C12 myotubular cells and have a moderate effect on grip strength, amino acid profile, and gene expression in C57BL/6 mouse skeletal muscle [126]. In an osteoporosis-induced mouse model, sEVs from dietary bovine milk enhanced bone health [127]. After incubating Raw264.7 cells with msEVs, tartaric acid phosphatase-resistant staining was significantly inhibited, indicating reduced differentiation of osteoclasts. The group of mice given sEVs had considerably higher bone mineral density. sEVs isolated from bovine milk may be potential candidates for preventing osteoporosis, improving bone remodeling, and inhibiting bone resorption.

#### 4.2.4. Promote Skin Regeneration

SEVs derived from stem cells usually have the function of tissue regeneration. More and more clinical studies use them as tissue regeneration materials to promote wound healing and reduce scar formation. Studies have shown that bovine colostrum also has excellent natural healing power, and its msEVs can promote the transformation of inflammation into tissue and further promote the healing of skin wounds [128], as shown in Figure 5B. msEVs can promote fibroblast proliferation, migration, and endothelial tube formation. In the mouse model of the excised wound, the msEVs can promote re-epithelialization, activate angiogenesis, and promote the maturation of the extracellular matrix. The mechanism may be that bovine msEVs can induce ECM deposition and regulate tissue regeneration by regulating the phosphorylation of the Smad pathway, and their miRNA-21 also plays a key regulatory role. It shows that they are very promising to be developed into anti-inflammatory drugs, especially for the treatment of skin wounds.

#### 4.2.5. Detection of Bovine Diseases

SEVs composition is finely controlled by the parental cells. Therefore, the analysis of sEV components can be used to infer the functional state of the parental cells or even the organism as a whole [1,129]. The analysis of multi-omics profiles or disease markers, including nucleic acids, proteins, and lipids, in msEVs can be used to determine the health of donor cows, laying the foundation for a non-invasive body fluid biopsy approach to detect disease [130]. In addition, msEVs also perform epigenetic modification functions. A profound understanding of sEV’s epigenetic modification functions is essential to improving breeding success and the profitability of dairy farms [131]. 

Staphylococcus aureus infection is a common disease in cattle. The variations in the sEV composition of cattle infected with S. aureus are more intensively studied. MiRNA content in msEVs is regulated by many variables, including the health status of the host and the stage of lactation. Analysis of staphylococcus aureus-infected bovine msEVs genomic broad miRNA profiling revealed significant expression differences between healthy and infected animals for at least 18 miRNAs, with up-regulation of miR-142-5p, miR-183, and miR-223 and down-regulation of miR-99a-5p, miR-101, and miR-2285-3p. Changes in miR-223, miR-142-5p, miR-378, and miR-185 are particularly thought of as promising possibilities for mastitis screening indicators [9,132]. Proteomic analysis can likewise provide much relevant information. Quantitative data revealed that among the 2971 proteins identified in msEVs, over 300 were associated with host defense. Additionally, compared to uninfected controls, 94 proteins were significantly differently regulated in cow’s msEVs from S. aureus-infected animals [133]. Western blotting data demonstrate that in msEVs from BLV-infected cows, the structural proteins gp51 and p24 of the bovine leukemia virus (BLV) were discovered [134].

#### 4.2.6. Other Functions

sEVs from milk taken orally reduce the signs and symptoms of arthritis in IL-1 Ra-deficient spontaneous polyarthritis mice and arthritis animal models generated by collagen [135]. In response to chemotherapeutic side effects, msEVs protect macrophages from cisplatin-induced cytotoxicity [136]. A protein known as butyryl protein (BTN), which shares an epitope with the myelin oligodendrocyte glycoprotein (MOG), was identified in milk, and transdermal administration of msEVs to patients with multiple sclerosis induced the development of MOG-specific tolerance for use in MOG-specific immunotherapy [137]. MsEVs can be an effective medicinal ingredient to improve skin lightening. MiR-2478 insEVs inhibits melanogenesis by the Akt-GSK3β signal pathway and is able to reduce melanin content, tyrosinase activity, and melanogenesis-related gene expression in melanoma cells and melanocytes [138]. The use of msEVs might become a potential way to treat cardiac fibrosis. MsEVs attenuate the effects of cardiac fibrosis by enhancing angiogenesis and improving cardiac function in rats with cardiac fibrosis [139]. Colostrum-derived sEVs have the potential to promote hair regeneration. It was reported that colostrum sEVs could promote dermal papillary cell (DP) cell proliferation, rescue dihydrotestosterone (DHT)-induced follicular development arrest, and induce dorsal hair regrowth in mice at levels similar to minoxidil therapies without related side effects, such as rash [140].

### 4.3. Potential Health Risks Associated with msEVs

There is a general belief that excessive intake of nutrients is extremely detrimental to health. Milk is popular for its excellent taste and nutritional content, but excessive intake is considered to be one of the contributing factors to adverse consequences such as diabetes, obesity, and cardiovascular disease. sEVs’ function in milk has also received a lot of interest. Epidemiological studies have linked msEV intake to a higher risk of type 2 diabetes, obesity, Parkinson’s disease, and cardiovascular disease [141,143,144,145]. The reason why msEVs may increase the risk of these diseases is often believed to be caused by the miRNAs they contain. For example, miR-148a is a biomarker of obesity in human subjects and mouse models and can inhibit Wnt1 and promote adipocyte differentiation [141]. Furthermore, miRNA-148a and miRNA-21 can enhance miRNA-148a/DNMT1 dependency. The α-Synuclein may further aggravate type 2 diabetes and Parkinson’s disease [143]. These concerns are warranted, but until more systematic studies or studies that can corroborate each other are available, remain positive about their use.

## 5. MsEVs as Drug Delivery Vehicles

Advancements in medication delivery technology are gaining momentum as lipid nanoparticles (LNPs) delivering mRNA are widely recognized as vaccines against COVID-19 outbreaks. The emergence of novel delivery systems may offer opportunities for diseases that are difficult to cure with existing drugs or methods. However, viral vectors and manufactured nanoparticles have obvious disadvantages as exogenous delivery vehicles, such as high toxicity, high immunogenicity, low targeting, a short half-life, easy clearance, and the production of antibodies after multiple doses that affect the delivery effect.

sEVs as a natural drug delivery system inherently perform functions such as signal delivery in vivo, do not cause the problems described above, and have much superior potential. These advantages include the small size of sEVs, their ability to penetrate deeper tissues, their slightly negative surface charge that contributes to long circulation, their similarity to cell membranes, and their deformable cytoskeleton [146,147,148]. Additionally, some sEVs show improved resistance to immune system destruction or clearance [149]. sEVs are not only highly biocompatible and less toxic and immunogenic, but also have a targeted “homing” effect. The ability of sEVs to reach all organs of the body after entering by intramuscular or intravenous routes is largely due to their capacity to penetrate past physiological barriers such as the placental, gastric, and blood-brain barriers. Drug resistance can be reduced by sEVs-mediated delivery because it can avoid the P-glycoprotein drug efflux pathway. sEVs have “do not swallow me” signaling molecules like CD47 on their surface, which resist macrophage phagocytosis and lengthen in vivo half-lives.

Oral administration is the preferred method of drug delivery compared to intramuscular, subcutaneous, and intravenous administration. However, the challenges of oral drug delivery are enormous, which is attributed to the gastrointestinal tract’s hostile environment rich in gastric acid and digestive enzymes, which makes it challenging for medications to enter the body through the stomach, resulting in low bioavailability. Therefore, the search for delivery systems that can be used for oral drug delivery is a relentless pursuit for researchers. 

Due to their unique evolutionary advantages over other sources of sEVs, msEVs are expected to be a new vehicle for oral drug delivery. The resistant glycoproteins (XDH, BTN, and MUC1) and surface proteins (FLOT1, ICAM1, ALIX, and EpCAM) of msEVs make them resistant to pepsin and have good stability [150]. Some studies have also shown that msEVs are absorbed by the intestine in the form of intact particles under the protection of Fc receptors (FcRn) [98]. 

### 5.1. Biosafety of msEVs

The safety of sEVs generated from milk as medicine delivery systems is the primary concern. Milk provides a large number of nutrients to the body, and humans have a long history of milk consumption, so it is widely believed that it is very safe to drink milk from cows. Due to the complexity of the raw material, it remains difficult to isolate pure sEVs from milk. Some proteins in milk are important allergens. Therefore, as an important component of milk, the biosafety and biocompatibility assessment of msEVs is indispensable.

Studies have revealed that high-purity msEVs are readily absorbed by macrophages without cytotoxic effects [45]. In addition, msEV injection into the tail vein did not result in systemic toxicity [45]. MsEVs did not produce any by-effects or immunogenicity in mice, and no anaphylactic shock effect was observed after continuous administration in mice [151]. All the mice survived the experiment in good health. Both single and repeated oral administrations of msEVs at a 25 mg/kg dosage led to no discernible changes in the clinical symptoms, indicating the safety of msEVs. Additionally, compared with the control group, oral administration of msEVs had no discernible effects on aspartate aminotransferase or creatinine levels in the serum [33]. This indicates that msEVs did not exert detrimental impacts on liver and kidney performance and were well tolerated.

### 5.2. Engineering of msEVs

Engineering sEVs is an important tool to expand their applications. sEVs are highly engineerable, and engineered sEVs are endowed with tissue or cell specificity, such as targeting. sEVs can be engineered in a manner that includes genetic approaches, biological manipulation, chemical and physical modifications, and other methods to engineer the surface and interior of sEVs [152].

#### 5.2.1. Cargo Loading 

Drug delivery in sEVs can be achieved by direct or indirect loading: (1) drugs with small molecules can be loaded directly into sEVs through passive incubation methods or active strategies like electroporation, saponification, repeated freeze-thawing, ultrasonication, extrusion, etc. These are the common methods for loading msEVs with drugs, followed by further purification to remove the unencapsulated small molecules; (2) indirect methods by modifying the sEV-derived cells, e.g., using transfection or transduction expression vectors, so that the sEV donor cells secrete sEVs containing functional drug molecules. In contrast, msEVs are mainly isolated and purified before being engineered and are currently focused on loading drugs into them for functional applications. 

Co-incubation, the simplest method of sEV drug loading, is appropriate for loading hydrophobic small compounds like curcumin and paclitaxel. The co-incubation of hydrophobic drugs with sEVs was loaded in a passive diffusion manner, but the co-incubation method of drug loading is less efficient. In contrast, loading of such substances as hydrophilic molecules and macromolecules requires transient perforation or disruption of the membrane because the sEVs are blocked by lipid membranes, and the methods that can be used include freeze-thaw cycles, electroporation, extrusion, ultrasound, and surfactant treatment.

The freeze-thaw cycling method is based on co-incubation and takes advantage of the process of disruption and remodeling of the membrane structure of sEVs in buffer for loading. The sEVs membrane is momentarily disrupted by the production of ice crystals during freezing, thus permitting hydrophilic substances to penetrate the interior before membrane reconstruction, and after thawing, the ice crystals disappear and the sEVs membrane undergoes remodeling and encapsulates the drug. The extrusion method involves mixing the sEVs with the free drug and passing it through channels containing nanoscale pores, where shear breaks the lipid membrane and permits the exogenous drug to enter the sEVs. Electroporation can form pores on the surface of the sEV membrane, and the pore can be closed, and the lipid layer can be formed again when the electric field is removed. Electroporation can be used to aid the entry of hydrophilic small molecules into sEVs and improve the loading efficiency of siRNA [30]. The development of continuous flow electroporation is expected to increase the scale of application of this method. Ultrasonication is another method for loading drugs into sEVs [153,154]. Ultrasound-based drug loading methods use an ultrasound probe to apply ultrasound energy, which reduces the rigidity of the sEV membrane and enables drug diffusion. However, ultrasound treatment may cause the cleavage of sEVs. Cargoes such as nucleic acids and chemicals can be penetrated into sEVs by ultrasound ultrasonication, but the cargoes may mostly only adhere to the surface of sEVs.

Recently, a team developed the “ENP” drug delivery system, a nanofluidic device containing 30,000 modules working in parallel and capable of high-throughput loading a variety of cargoes into sEVs. The sEVs pass through nanochannels that are similar in size to them, and mechanical compression and fluid penetration have the ability to shear the sEV membrane, allowing cargo molecules to enter the sEVs while preserving their integrity. The sEVs nanopuncturer uses an array of 30,000 nanochannels, the highest number of channels available for a single chip. sEVs treated with the sEVs nanopuncturer can deliver drugs to human non-small cell lung cancer cells and can lead to their apoptosis [155].

The drug can also be loaded into sEVs using CaCl_2_. This method was carried out by sEVs and cargo in 100 mm CaCl_2_ and subjected to ice incubation and thermal excitation treatment [156]. The load efficiency of this method is close to that of electroporation. Saponin is a kind of decontaminant that can open the pores of the lipid membrane by removing membrane cholesterol. The disadvantage of saponins is their potential cytotoxic effect. 

Cationic lipids can also be used to deliver mRNA into sEVs, and the method has been shown to be safe and has been applied to create the SARS-CoV-2 vaccine [157]. In this method, the mRNA was mixed with cationic lipids to form polymers, and then sEVs were added for further fusion to achieve the purpose of loading. Lipofectamine uses cationic substances to facilitate the interaction with the lipid membrane and subsequent internalization, which can effectively load nucleic acids into the sEV’s interior [158]. Exogenous hsa-mir148a-3p could be loaded into msEVs by Lipofectamine 2000, and its biological function was evaluated [70].

#### 5.2.2. Modification of msEVs

SEVs of different origins are often targeted with similar “homing” effects. sEVs have high engineering ability, and surface-engineered sEVs can achieve cell and tissue specificity and similar “homing” effects. For example, synthetic biology can be used to add biomolecules (targeting ligands, immune evasion molecules, and stimuli-responsive molecules) to the sEV surface.

Targeted delivery of drugs through sEVs raises the medicinal agent’s local concentration and reduces adverse effects. The surface-modified msEVs can achieve the purpose of specific drug delivery to tumor cells. Loading the iRGD tumor-targeting peptide on the sEVs surface could change the organ aggregation rate of sEVs [98]. Hyaluronic acid-modified sEVs could target delivering doxorubicin (DOX) to tumor cells with high expression of CD44, thus triggering significant tumor cell death [159]. The approach involves functionalizing hyaluronic acid (HA) with the amphiphilic molecule DSPE-PEG2000, which allows Dox-loaded msEVs to be spontaneously decorated with HA on a phospholipid bilayer. Folic acid (FA), a tumor-targeting ligand, was linked to msEVs and loaded with paclitaxel (PAC), which increased the cancer cell targeting ability of sEVs, thereby enhancing the tumor-killing effect. Subcutaneous tumor xenografts were significantly inhibited by oral FA-ExoPAC, but a similar dose of PAC has no obvious effect [160]. The HA-msEVs-FA system constructed with msEVs modified by FA-encapsulated CD44 specific ligand HA could fight against liver fibrosis by regulating NLRP3-mediated apoptosis [161].

MsEVs with environmentally responsive functions are also being developed. The modified msEVs were used to target the transport of Adriamycin to triple-negative breast cancer cells and were able to respond intelligently to oxygen concentration [162]. The peptide-targeted hypoxia-responsive msEVs were created in this experiment using Neurofibrillin receptor agonist peptide (iRGD) and hypoxia-responsive liposomes. These doxorubicin-encapsulated sEVs in a model of triple-negative breast cancer cell monolayers and three-dimensional (3D) spheres demonstrated their environmentally responsive tumor-killing ability.

Surface modification of sEVs can improve the half-life of msEVs in vivo. In one study, msEVs were hydrophobically coated with a hydrophilic polyethylene glycol (PEG) layer, which greatly decreased the breakdown of sEVs in an acidic stomach environment and increased the permeability by more than 3-fold [72]. CD47 is a “do not swallow me” signal, an immune escape indicator abundant on the surface of cancer cells. By attaching to SIRP, CD47 reduces macrophages’ capacity to phagocytose tumor cells [163]. Many sEVs also contain CD47 signaling proteins, which prolong the half-life of sEVs circulating in vivo. Engineering donor cells or directly modifying sEVs to increase the abundance of CD47 signaling on their surface is becoming an important direction for engineering sEVs.

### 5.3. MsEVs for Drug Delivery 

**Figure 6:**
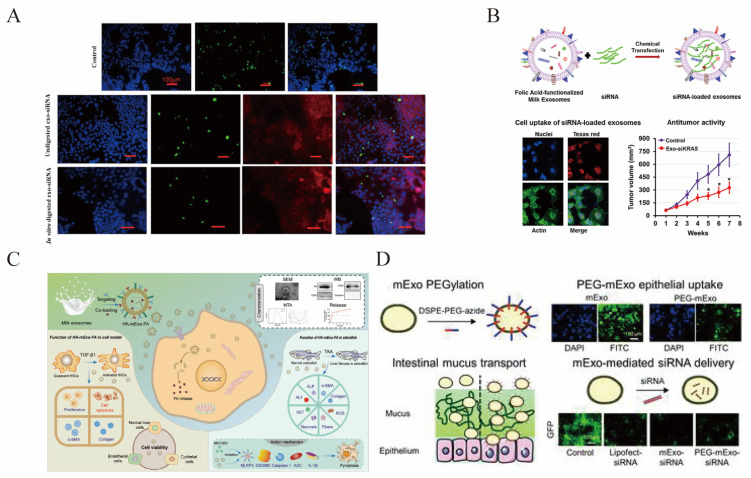
MsEVs are developed as a drug delivery system. (**A**) Delivery of AF-488 siRNA in Caco-2 cells via msEVs in vitro; Copyright 2017, American Chemical Society [170]. (**B**) MsEVs deliver siRNA for the treatment of cancer; Copyright 2019, Elsevier [171]. * *p* < 0.05. (**C**) CD44 targeting the drug delivery system of sEVs loading FA for treatment of liver fibrosis; Copyright 2023, John Wiley and Sons [161]. (**D**) MsEVs with enhanced mucus penetrability for oral delivery of siRNA; Copyright 2020, Royal Society of Chemistry [72].

MsEVs can be utilized as efficient drugs and gene carriers, as shown in Figure 6. Due to their distinct evolutionary capacity and distinctive characteristics, sEVs obtained through acid administration and ultracentrifugation are tolerable in vivo, and msEVs may travel through the gastrointestinal tract and be completely absorbed as entire particles. The drugs are encapsulated in sEVs and avoid decomposition by the gastrointestinal environment [58,151,164]. Bioluminescence experiments in mice by the oral route confirmed that sEVs have high bioavailability [98]. sEVs therefore have a significant potential for being efficient medication carriers.

#### 5.3.1. Delivery of Chemical Drugs

Several chemotherapeutic agents exhibiting various functions, such as superior antioxidant, antiproliferative, anti-inflammatory, and pro-apoptotic, are being developed. However, their development is limited by their low permeability, low solubility, and poor oral bioavailability. Hence, it is necessary to develop efficient delivery systems and systems with robust bioavailability. MsEVs are widely recognized as chemotherapeutic agent carriers and have many advantages in oral administration. Using the proper solvents, a variety of chemotherapy drugs were loaded into msEVs, including curcumin, anthocyanins, paclitaxel, celastrol, rotenone, and doxorubicin [33,154,165,166,167]. Due to the differences in molecular weight, lipophilicity, and functional groups of these small molecules, the efficiency of sEV loading is between 10% and 40%.

When loaded into sEVs, curcumin could increase intestinal permeability and withstand breakdown by human digestive enzymes [166]. sEVs are agents of anthocyanins that are able to fight against many cancer types. Compared to free Anthos, Anthos-loaded sEVs have enhanced anti-inflammatory and anti-proliferative functions on a variety of cancer cells in vitro and showed significant enhancement of lung cancer xenograft therapy. In wild-type mice, anthos-loaded sEVs exhibited no obvious or systemic toxicity symptoms [168]. MsEVs loaded with anthocyanins can prevent colorectal cancer and alleviate symptoms caused by an intestinal microbiota imbalance [169]. Paclitaxel is traditionally administered by intravenous injection. Due to the poor solubility of paclitaxel in water, oral administration cannot reach the therapeutic dose. MsEVs encapsulated with paclitaxel could overcome these problems by oral administration [165]. It was demonstrated that msEVs carrying paclitaxel showed good and stable antitumor efficacy [165]. Furthermore, msEVs carrying paclitaxel reduced hepatic, renal, and systemic toxicity [165]. sEVs offer an effective alternative to oral administration of chemotherapeutic agents with high efficacy, low cost, and high safety.

#### 5.3.2. Delivery of Nucleic Acids

MsEVs are attracting delivery vectors for small interfering RNA (siRNA) [72,170,171], as shown in Figure 6A,B,D. SiRNA has gene silencing functions and can treat various diseases, including cancer. However, there are many bottlenecks in siRNA development, including stable intracellular delivery and targeted delivery. sEVs can stably and efficiently deliver siRNA to specific receptor cells. Encapsulating siRNA in msEVs can resist harsh digestive processes, including saliva, gastric juice, and bile, improve intestinal permeability, and enhance payload protection [170,171,172]. β-Catenin siRNA was packed into msEVs by using lipofectamine, and these sEVs showed good target gene knockdown efficiency [172]. Using msEVs loaded with siRNAs targeting VEGF, EGF, AKT, MAPK, and KRAS, it showed a 2- to 10-fold decrease in the expression levels of the corresponding targets in various cancers [171], as shown in Figure 6B. When Bcl-2 siRNA was loaded into msEVs using an ultrasonic technique, both in vitro and in vivo cancer cell migration and the formation of gastrointestinal tumors were significantly suppressed [173].

MsEVs are also good miRNA carriers. The miRNA hsa-mir148a-3p, which was exogenously loaded into msEVs, could be ingested by HepG2 and Caco-2 cell lines, and its biological function was explored by microarray gene expression [70]. Therefore, msEVs can be an important source of functional miRNA nanocarriers and help develop new methods of nucleic acid therapy.

#### 5.3.3. Delivery of Other Small Molecules

MsEVs-encapsulated epigallocatechin gallate (ECG) enhances neuroprotection against Parkinson’s disease [154]. MsEVs were successfully delivered to SHSY5Y cells and inhibited rotenone-induced SHSY5Y cell injury through anti-apoptotic and anti-phagocytosis [154]. Evidence showed that the anti-diabetic drug liraglutide, loaded into msEVs, could reduce blood glucose levels when administered sublingually [174].

## 6. MsEVs and Nutritional Supplements

Mammalian breast milk itself has great nutritional value and is capable of performing physiological functions such as immune enhancement, resistance to bacterial infection, and resistance to oxidative stress. A large part of these functions may be a direct function of the proteins, nucleic acids, lipids, and sugars carried by their sEVs. Breastfeeding is associated with a lower risk of infection, immune-mediated disorders, obesity, and even cancer during the first six months of life and can satisfy all of an infant’s nutritional demands at various stages of growth [175]. Infants who are breastfed have higher Preschool Language Measure Scores, the Psychomotor Developmental Index, and the Mental Developmental Index than infants who are fed different formulas, according to phenotypes seen in infant feeding studies [59].

The physiological functions performed by sEVs provide strong evidence to promote breastfeeding in infants. However, a large number of infants and children require the use of deeply processed infant formula or other dairy products as nutritional supplies due to the deficiency of maternal breast milk. Key elements such as sEVs are often lacking in infant formula or commercially available deeply processed dairy products, or functional factors are disrupted during production. EVs structural integrity and molecular makeup are impacted by conventional industrial processing of milk [176]. Commercial milk heated by pasteurization or ultra-high-temperature sterilization contains fewer or no intact sEVs [177]. Initial evidence suggested that msEVs were bound by the lectins in the infant soy formula, resulting in reduced absorption [178]. Compared to breast milk, the amount of sEVs and miRNAs in infant formula is negligible. Some highly conserved miRNAs exist in human, bovine, and goat milk but cannot be detected in infant formula [179].

sEVs can be added to infant formula or other health foods for the development of new clinical therapies. MsEVs exhibit good cross-species tolerance and do not cause unfavorable immunological or inflammatory reactions. The development of msEVs as an important functional component in this field is still in its infancy. It is also possible that the msEVs miRNA concentration is not sufficient to produce gene regulation [180]. Large amounts of exogenous miRNAs or other regulatory molecules can be engineered to be loaded into msEVs, and the oral msEV-loaded cargo can reach tissues in the body. There may be great efficacy and market prospects for adding msEVs to infant formulas or nutraceuticals targeting the elderly population.

## 7. Conclusions and Prospects

The phospholipid bilayer, proteins, and nucleic acids of msEV are very similar to those of their parent cells, and their compositions are finely controlled by parent cells. Thus, msEVs can be employed as biomarkers to detect diseases. Furthermore, msEVs are involved in regulating multiple biological processes, including immunoregulation, anti-bacterial infection, and antioxidants, which play a beneficial role in human health at multiple levels, such as intestinal health, bone and muscle metabolism, and microbiota regulation. In addition to directly utilizing the natural functions of msEV, due to its superior biological activity over LNP, msEV can also serve as an important carrier for delivering exogenous substances. Although the application prospect of msEVs is promising, there is still much work to be done in the commercial application of biopharmaceuticals.

Before the practical application of msEVs, it is necessary to overcome the challenge of large-scale purification. In the biological products industry, utilizing raw materials from animal sources is usually restricted. In daily life, many people are allergic to dairy products. This puts forward higher requirements for the development of msEVs. Improving the level of purification and reducing the residue of allergic substances are two important solutions to this problem. The recent emergence of new technologies such as microfluidics has provided new ideas for the purification of msEV. However, for the large-scale application of msEVs, microfluidic methods still require some optimization.

Through histological analysis, it was confirmed that msEVs are rich in active substances such as nucleic acids, proteins, carbohydrates, lipids, etc. Their content varies depending on season, species, physiological conditions, and other factors and has a significant impact on physiology and pathophysiology. There may also be significant heterogeneity in msEVs from different production batches. In order to obtain a unified-quality msEV, the following issues may need to be considered: 1. A stable and reliable milk source supply is required; 2. the establishment of a raw milk quality control system; 3. the establishment of standardized production operation processes; and 4. the establishment of strict quality standards for MSEV, for example, using several key proteins or miRNAs to establish product release standards.

The main application pathway for msEVs is oral administration. After passing through the gastrointestinal barrier, they mainly accumulate in the liver, and some of them carry miRNAs and other substances through the blood circulation to other organs for action. There is relatively little research on the natural targeting of msEVs, and most of it focuses on engineering modifications to improve their targeting. In addition to the engineering modification of msEVs for loading exogenous substances and improving targeting, it can also achieve an extension of their half-life. In addition, the application of msEVs in nasal sprays or injection preparations can be discussed. In order to address these issues, it is necessary to cultivate more msEVs professionals, improve and develop large-scale purification and engineering technologies, and establish industry standards to promote the commercialization of products. In the near future, it is believed that both natural msEV functional products and engineered products will be clinically transformed.

## Figures and Tables

**Table 1:** sEVs common protein markers.

sEVs Proteins	MW (kDa)	Classification	Function	Specimen Source
CD63	63	Four transmembrane proteins	It is a lysosomal membrane protein with the activity of activating platelet surface antigens;	Bovine milk [34,36,37], mesenchymal stromal cells [38], and plasma [39,40];
CD9	24–27	Four transmembrane proteins	Participate in the interaction between cells and the outside world;	Bovine milk [36], mesenchymal stem cells [41,42], and saliva [43,44];
CD81	81	Four transmembrane proteins	Key structural sites for perceiving external signals in cells;	Bovine milk [37,45], synovial fluid [46], and cerebrospinal fluid [47];
TSG101	44	Internal signature proteins	A component of the functional ESCRT-I complex that regulates vesicular transport;	Bovine milk [34,48], rat serum [40], and bile [49];
ALIX	95	Internal signature proteins	A phylogenetically conserved cytosolic scaffold protein;	Bovine milk [34,36,48], brain [50], and fibroblast [51];
HSP70	70	Internal signature proteins	It is an important member of the heat shock protein family.	Bovine milk [34,45], plasma [39,44], and urine [52,53].

**Table 2:** Common methods of sEV separation.

Separation Method	Principle	Purity	Production	Time Consuming	References
PEG precipitation purification	Macromolecule aggregation precipitation;	+	+++	+	[57]
Ultracentrifugation	Difference of sedimentation coefficient;	++	++	++	[53,58,59]
Density gradient centrifugation	Density gradient difference;	+++	+	+++	[60]
Ultrafiltration centrifugation	Specific molecular weight cutoff;	++	+++	+	[61]
Tangential Flow Filtration	Tangential filtering;	++	+++	+	[62]
Size exclusion chromatography	Particle size difference;	+++	++	++	[63,64,65]
Affinity purification	Intermolecular specific binding.	++++	+	++	[66]

Note: The “+” in the table represents different degrees, and the more “+”, the higher the degree.

**Table 3:** Functions of msEVs.

Function	Therapeutic Agent	Effect	Mechanism	References
Immunoregulation	TGF-β and miRNA-30b	Play a crucial role in the biogenesis and improvement of infant immune function.	Regulation of immune-related factors (such as miRNAs and antibodies).	[100,101,102,103,104,105,106]
Regulation of intestinal tract function	bta-miR-34a, miR-155, and miR-146a	Antioxidant stress; Resistance to hypoxic injury; Reduce the inflammatory response induced by LPS, DSS, and other factors in the mouse intestinal model.	Enhance cell activity, inhibit inflammation, regulate intestinal flora, etc.	[107,108,109,110,111,112,113,114,115,116,117,118,119,120,121,122,123]
Development of muscle and bone	miR-21 and miR-29a	The growth and development of muscle and bone can be altered by miRNAs from msEVs.	Increase the number of osteoblasts, promote bone formation and osteoblast differentiation, and encourage myofiber formation in myotube cells.	[124,125,126,127]
Promote skin regeneration	TGF-β and miRNA-21	Promote the transformation of inflammation into tissue and further promote the healing of skin wounds.	Induce ECM deposition and regulate tissue regeneration by regulating the phosphorylation of the Smad pathway.	[128]
Detection of bovine diseases		Bovines can be monitored for infection with pathogenic bacteria such as Staphylococcus aureus or their health status.	Examining the nucleic acids, proteins, and lipids in msEVs to identify illness signs.	[129,130,131,132,133,134]
Alleviates arthritis symptoms	immunoregulatory microRNAs (miR-30a, miR-223, miR-92a), beta-lactoglobulin mRNA, and milk-specific beta-casein	Delayed the onset of arthritis, and histology showed diminished cartilage pathology and bone marrow inflammation in both models.	Decreased MCP-1 and IL-6 production by splenic cells in serum; Decreased splenic Th1 (Tbet) and Th17 (RORT) mRNA levels, which were also associated with decreased anticollagen IgG2a levels.	[135]
Reduce the side effects of chemotherapy		Protect cells from chemotherapeutic drug-induced cytotoxicity.	Affect the cell cycle of RAW 264.7 with and without cisplatin.	[136]
Use in MOG-specific immunotherapy	butyrophilin (BTN)	Could be thought of as an attractive method to help patients with multiple sclerosis develop MOG-specific tolerance.	The BTN content of these vesicles can pass past the skin’s epidermis and other biological barriers, serving as Trojan horses for the body.	[137]
Can be a useful medicinal ingredient for improving skin lightening	miR-2478	Inhibit melanin production.	Through the Akt-GSK3 signaling pathway, Rap1a expression inhibition reduced melanogenesis.	[138]
Might become a potential way to treat cardiac fibrosis		Improved heart performance in cardiac fibrosis rats.	Significant improvements were made to the proangiogenic growth factors.	[139]
Have the potential to promote hair regeneration.		Promote hair regeneration.	Promote dermal papillary cell (DP) proliferation and rescue dihydrotestosterone (DHT)-induced follicular development arrest.	[140]

## Data Availability

No additional data are available.

## Abbreviations

BLV	Bovine leukemia virus
BTN	Butyrophilin
DAI	Disease activity index
DNMT1	DNA methyltransferase 1
DSS	Dextran sulfate sodium salt
DOX	Delivering doxorubicin
DOX	Delivering doxorubicin
DSPE	1,2-distearoyl-sn-glycero-3-phosphoethanolamine
DP	Dermal papillary cell
DHT	Dihydrotestosterone
ECG	Epigallocatechin gallate
ENP	Exosome nanopuncturer
ESCRT	Endosomal sorting complex required for transport
FA	Folic acid
HA	Hyaluronic acid
LNPs	Lipid nanoparticles
lncRNA	Long non-coding RNA
LPS	Lipopolysaccharide
msEVs	Milk-derived small extracellular vesicles
miRNA	MicroRNA
mRNA	Messenger RNA
ncRNA	Non-coding RNA
NEC	Necrotizing enterocolitis
PAC	Paclitaxel
PEG	Polyethylene glycol
sEVs	Small extracellular vesicles
SEC	Size-exclusion chromatography
siRNA	Interfering RNA
SAM	Severe acute malnutrition
TFF	Tangential flow filtration
TLR4	Toll-like receptor 4
